# Estrogen deficiency impairs integrin α_v_β_3_-mediated mechanosensation by osteocytes and alters osteoclastogenic paracrine signalling

**DOI:** 10.1038/s41598-019-41095-3

**Published:** 2019-03-15

**Authors:** Ivor P. Geoghegan, David A. Hoey, Laoise M. McNamara

**Affiliations:** 1 Mechanobiology and Medical Device Research Group (MMDRG), Biomedical Engineering, National University of, Ireland Galway, Ireland; 2 Centre for Research in Medical Devices (CÚRAM), National University of Ireland, Galway, Ireland; 3 Trinity Centre for Bioengineering, Trinity Biomedical Sciences Institute, Trinity College Dublin, Dublin 2, Ireland; 4 Department of Mechanical and Manufacturing Engineering, School of Engineering, Trinity College Dublin, Dublin 2, Ireland; 5 Advanced Materials and Bioengineering Research Centre, Trinity College Dublin & RCSI, Dublin 2, Ireland

## Abstract

The integrin α_v_β_3_ has been shown to play an important role in osteocyte mechanotransduction. It has been reported that there are fewer β_3_ integrin-containing cells in osteoporotic bone cells. Osteocytes cultured *in vitro* under estrogen deficient conditions demonstrate altered mechanotransduction. However, it is unknown whether the altered mechanotransduction in estrogen deficient osteocytes is directly associated with defective α_v_β_3_ expression or signalling. The objective of this study is to investigate the role of estrogen deficiency for regulating MLO-Y4 cell morphology, α_v_β_3_ expression, focal adhesion formation and mechanotransduction by osteocytes. Here, we report that estrogen withdrawal leads to a smaller focal adhesion area and reduced α_v_β_3_ localisation at focal adhesion sites, resulting in an increased *Rankl*/*Opg* ratio and defective *Cox-2* responses to oscillatory fluid flow. Interestingly, α_v_β_3_ antagonism had a similar effect on focal adhesion assembly, *Rankl*/*Opg* ratio, and *Cox-2* responses to oscillatory fluid flow. Taken together, our results provide the first evidence for a relationship between estrogen withdrawal and defective α_v_β_3_-mediated signalling. Specifically, this study implicates estrogen withdrawal as a putative mechanism responsible for altered α_v_β_3_ expression and resultant changes in downstream signalling in osteocytes during post-menopausal osteoporosis, which might provide an important, but previously unidentified, contribution to the bone loss cascade.

## Introduction

Osteocytes are the most abundant cells in bone and are responsible for mediating the balance between bone formation and resorption^1^. It has been proposed that osteocytes detect mechanical stimuli using mechanosensitive proteins, include stretch activated ion channels, gap junctions, primary cilia and integrins^2–4^ and transduce them into biochemical responses^5,6^. Molecular factors produced by osteocytes regulate osteoclasts and osteoblasts, in particular RANKL and sclerostin, which promote osteoclast formation and inhibit osteoblastogenesis respectively, and OPG, which acts as a decoy receptor for RANK and thereby prevents osteoclast formation^7–10^.

Integrins are heterodimeric transmembrane proteins, comprised of α and β subunits, which connect the intracellular cytoskeleton to the extracellular matrix through protein complexes known as focal adhesions (FA), which also comprise proteins such as vinculin, α-actinin, talin, and paxillin^11,12^. Focal adhesions are involved in Focal Adhesion Kinase (FAK) and shc signalling^13,14^ and are widely understood to play a role in mechanosensation for many distinct cell types^15–21^. Osteocytes express both β_1_ and β_3_ integrins^4,11^ and it has been shown that β_1_ integrins localise around osteocyte cell bodies, whereas osteocyte cell processes have α_v_β_3_ integrins and both interact with the surrounding pericellular matrix^4,22^. It has been proposed integrin based adhesions and pericellular matrix tethers together facilitate strain amplification^4,23–25^. *In vitro* studies have shown that Ca^2+^ response to a fluid stimulus was highly polarised along osteocyte cell processes but this Ca^2+^ response was compromised when cultured with a small molecule inhibitor of α_v_β_3_^17^. Moreover, when α_v_β_3_ was blocked using an antagonist, *Cox-2* expression and PGE_2_ release were reduced and cell morphology was altered, whereby the cells had a reduced cell area and fewer cell processes^15^.

Post-menopausal osteoporosis is a disease characterised by a decrease in circulating estrogen levels and an imbalance in bone cell remodelling, which causes bone loss and an increased susceptibility to fracture^26^. Estrogen acts as a regulator to maintain the balance of osteoblasts and osteoclasts^27^ and enhances the response of bone cells to mechanical stress^28^. It has been reported that the estrogen receptors, ERα and ERβ, play a role in this mechanobiological response by osteoblasts and osteocytes^29,30^. In osteoblasts, estrogen was shown to increase *Opg* expression and augment *Cox-2* (via β_1_ integrins and ERs) response to fluid shear stress^31,32^ and decrease *Rankl* and *Sost* expression^33,34^. In osteocytes, supraphysiological levels of estrogen (100 nM) were shown to have a protective role against apoptosis^35,36^, to induce an intracellular Ca^2+^ response^37^ and increase connexion 43 gap junction expression and mechanosensitivity^38^. *In vitro* supplementation of culture media with levels of estrogen (10 nM) within the range of estrogen in healthy humans (pre-menopausal), was shown to led to increased osteogenic signalling by MLO-Y4 osteocytes^39^. However, most *in vitro* studies of osteocyte biology use culture media without exogenous estrogen, and thus there is a limited understanding of pre-menopausal levels of estrogen on osteocyte biology.

Human osteoblastic bone cells derived from osteoporotic patients have been shown to exhibit an impaired biochemical response (PGE_2_) to mechanical stress compared to those derived from healthy patients^40^. Estrogen deficiency *in vitro* can be achieved by pre-treatment with 17β-estradiol followed by estrogen withdrawal or addition of an estrogen receptor antagonist^39,41^. Estrogen withdrawal in osteocytes was shown to attenuate fluid flow-induced intracellular calcium signalling, thus altering osteocyte mechanosensitivity^39^, and lead to higher levels of osteocyte apoptosis, compared to estrogen treated cells^41^. Estrogen deficiency induced by ovariectomy (OVX) has been shown to lead to an altered tissue composition and mineral distribution within bone, altered mechanical environment of osteocytes and a reduction in β_3_-positive cells in cortical bone compared to controls^42–44^. However, it is unknown whether such changes arise as a direct response to reduced estrogen or the ability of the β_3_ integrins to facilitate mechanotransduction.

In this study, we test the hypothesis that altered osteocyte mechanosensitivity during estrogen deficiency is associated with an impairment in the mechanotransduction by β_3_ integrins. Specifically, we investigate (1) the role of pre-menopausal levels of estrogen for regulating MLO-Y4 cell morphology, focal adhesion formation and mechanotransduction response to fluid flow, (2) changes in α_v_β_3_ expression and spatial organisation in osteocytes during estrogen deficiency, and (3) whether altered mechanosensitivity of osteocytes under estrogen deficiency correlate to defective α_v_β_3_ expression and functionality.

## Results

### Estrogen supplementation, to mimic pre-menopausal levels, leads to more mature FA assembly and actin cytoskeleton, and increased Opg expression compared to osteocytes cultured in standard media

*In vitro* studies of MLO-Y4 osteocyte biology commonly use α-MEM culture media without the addition of estrogen. However, *in vivo* estrogen is important for normal bone cell function and so, to more closely mimic the physiological environment of osteocytes *in vivo*, in this study we first investigated the effect of supplementing culture media with pre-menopausal levels of estrogen (10 nM) on MLO-Y4 osteocytes under static and oscillatory flow conditions *in vitro*.

**Figure 1:**
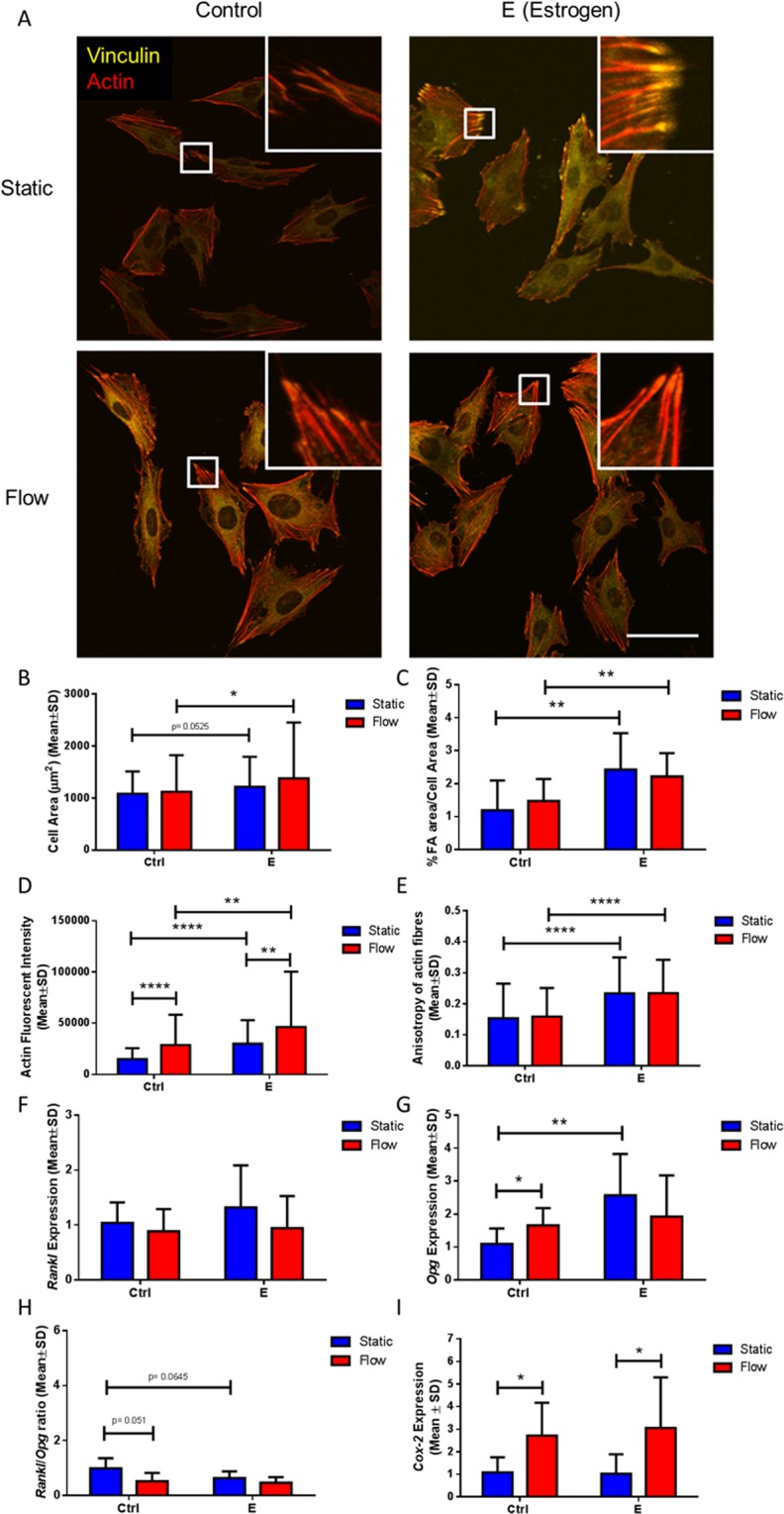
The effect of estrogen treatment and oscillatory fluid flow on MLO-Y4 cell morphology and downstream signalling. (**A**) Immunocytochemistry images showing actin fibres and vinculin staining (N = 3, n ≥ 111 cells per group). Quantification of the images showing (**B**) cell area, (**C**) % focal adhesion area/cell area, (**D**) actin fluorescent intensity, and (**E**) anisotropy of actin fibres. RT-PCR results of (F) *Rankl* expression (N = 5–10), (G) *Opg* expression (N = 7–10), (**H**) *Rankl*/*Opg* ratio (N = 5–7), and (I) *Cox-2* expression (N = 7–8) (Student’s t-test, *p < 0.05, **p < 0.01, ****p < 0.0001).

A robust cytoskeleton with distinct focal adhesion sites can be seen in both control and estrogen treated cells under static culture conditions (Fig. 1A). Quantification of these images showed that estrogen supplementation leads to the development of larger cells (p = 0.0525), which have more focal adhesions (p < 0.05) and most interestingly, these cells also have a significantly larger focal adhesion area (p < 0.01) (N = 3, n ≥ 111 cells per group), see Figs 1B,C and S1A. The actin fluorescence intensity was greater and the cells exhibited a higher degree of organisation (anisotropy) of the actin cytoskeleton in estrogen treated cells compared to controls (p < 0.0001 and p < 0.0001 respectively, N = 3, n ≥ 111 cells per group) (Fig. 1D,E). Moreover, estrogen treated osteocytes were shown to express higher levels of *Opg* (p < 0.01, n = 7–10), but *Rankl* expression was unaffected, and this ultimately resulted in a lower *Rankl*/*Opg* ratio (p = 0.0645, N = 5–7) (Fig. 1F–H). There was no difference in *Cox-2* expression in the estrogen treated group compared to controls (Fig. 1I). Thus, estrogen supplementation leads to larger osteocytes with more mature FA assembly and a more robust actin cytoskeleton, and these changes are accompanied by a reduction in gene expression associated with osteoclast differentiation (*Rankl*/*Opg*).

The application of oscillatory fluid flow resulted in an increase in actin fluorescent intensity for both control and estrogen treated cells, when compared to their static counterparts (p < 0.0001 and p < 0.01 respectively, N = 3, n ≥ 111 cells per group). However, fluid flow had no effect on cell area, focal adhesion area, or actin anisotropy for either cohort. Fluid shear stress led to a higher *Opg* expression in the control group (p < 0.05, N = 7–10), but had no effect on *Rankl* expression, and therefore, led to a lower *Rankl*/*Opg* ratio in that same group (p = 0.051, N = 5–7). There was no effect of fluid shear stress on *Rankl* or *Opg* expression in the estrogen treated group. Both control and estrogen treated cells showed a higher *Cox-2* response to fluid shear stress (p < 0.05 and p < 0.05 respectively, N = 5–7).

In summary, treatment of osteocytes *in vitro* with endogenous levels of estrogen, to mimic the pre-menopausal status of osteocyte biology, significantly alters cell behaviour, resulting in a more established cytoskeleton and a shift towards a more osteogenic phenotype when compared to widely used culture media for studies of MLO-Y4 osteocyte biology. Therefore, the estrogen treated groups were used as a control for studies of estrogen withdrawal, which are presented hereafter.

### Estrogen withdrawal alters osteocyte morphology, and this affect is further augmented by mechanical stimulation

Next we wished to understand what role estrogen withdrawal had on osteocyte morphology under both static and mechanical loading conditions.

**Figure 2:**
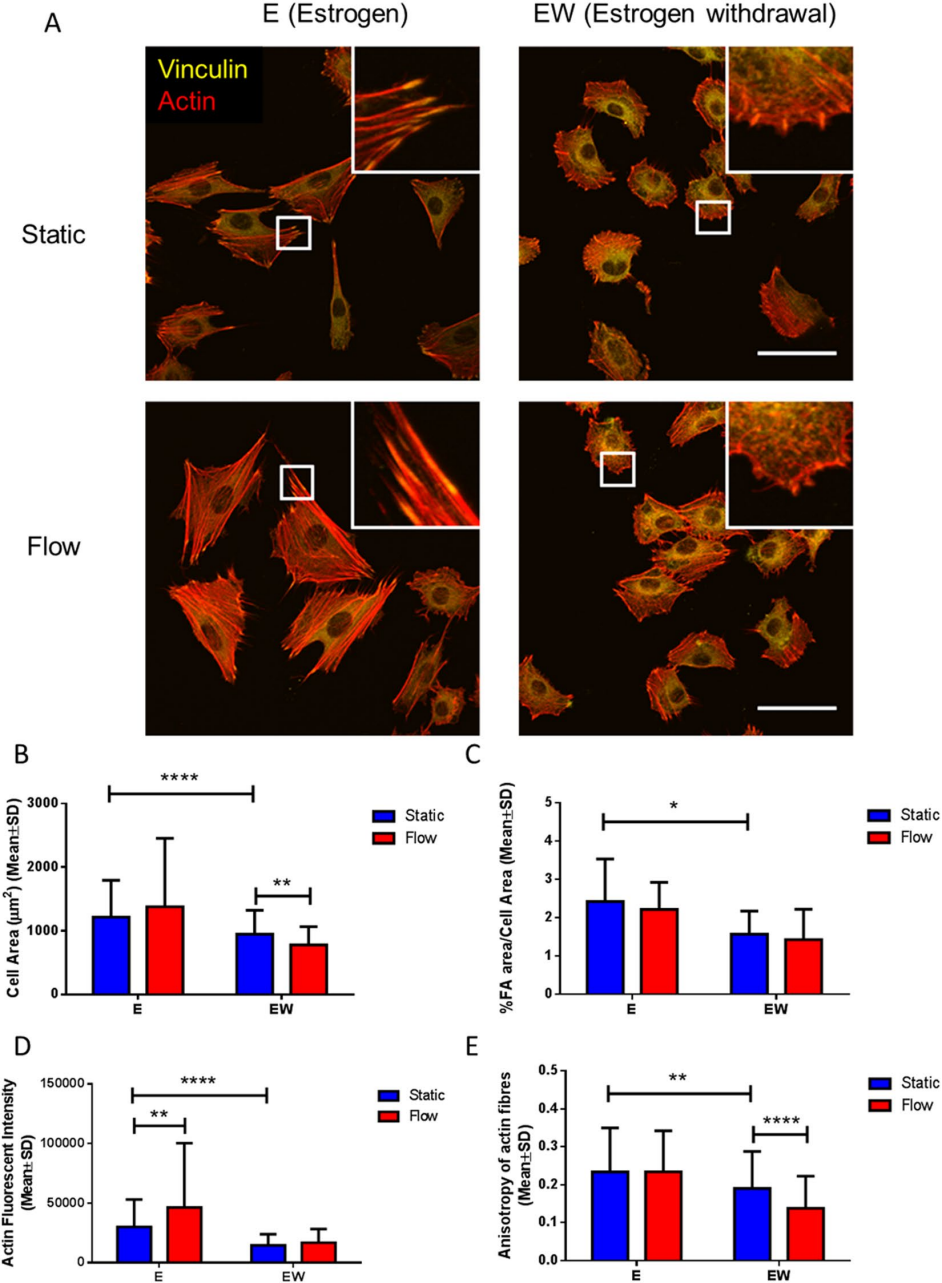
The effect of estrogen withdrawal and oscillatory fluid flow on MLO-Y4 cell morphology. (**A**) Immunocytochemistry images showing actin fibres and vinculin staining (N = 3, n ≥ 115 cells per group). Quantification of the images showing (**B**) cell area, (**C**) % focal adhesion area/cell area, (**D**) actin fluorescent intensity and (**E**) anisotropy of actin fibres. (Student’s t-test, *p < 0.05, **p < 0.01, ****p < 0.0001).

Under static conditions, cells cultured under estrogen withdrawal were observed to be smaller by immunostaining, and while they had focal adhesion sites, these were disordered, which was in contrast to estrogen treated cells that had distinct focal adhesion sites (Fig. 2A). Quantitative analysis of the actin and vinculin staining confirmed that cells cultured under estrogen withdrawal had a reduced cell and focal adhesion area, when compared to cells cultured under continuous estrogen conditions (p < 0.0001 and p < 0.05 respectively, N = 3, n ≥ 115 cells per group) (Fig. 2B,C). The actin staining in the estrogen withdrawal cells was less intense and there were fewer stress fibres, when compared to the estrogen treated cells, which had noticeable and numerous stress fibres (Fig. 2A). Quantitative analysis of the actin staining confirmed that the intensity and anisotropy of the actin fibres were lower following estrogen withdrawal in comparison to estrogen conditions (p < 0.0001 and p < 0.01 respectively, N = 3, n ≥ 115 cells per group) (Fig. 2D,E), demonstrating a less robust and ordered actin cytoskeleton for estrogen deficient osteocytes under static conditions.

Fluid shear stress led to a lower cell area in estrogen withdrawal cells when compared to its static counterpart (p < 0.01, N = 3, n ≥ 115 cells per group), but there was no difference in cell area or focal adhesion area following flow in the estrogen supplemented cells. Oscillatory fluid flow led to more intense actin staining in estrogen cells, compared to static groups (p < 0.01, N = 3, n ≥ 115 cells per group), but this was not observed for estrogen withdrawal cells, which also exhibited had a lower anisotropy following fluid flow (p < 0.0001, N = 3, n ≥ 115 cells per group), indicating a less ordered actin fibre organisation. Taken together, this data points to a defect in the formation of mechanosensitive focal adhesion sites following estrogen withdrawal.

### Estrogen withdrawal resulted in reduced α_v_β_3_ localisation at focal adhesion sites

**Figure 3:**
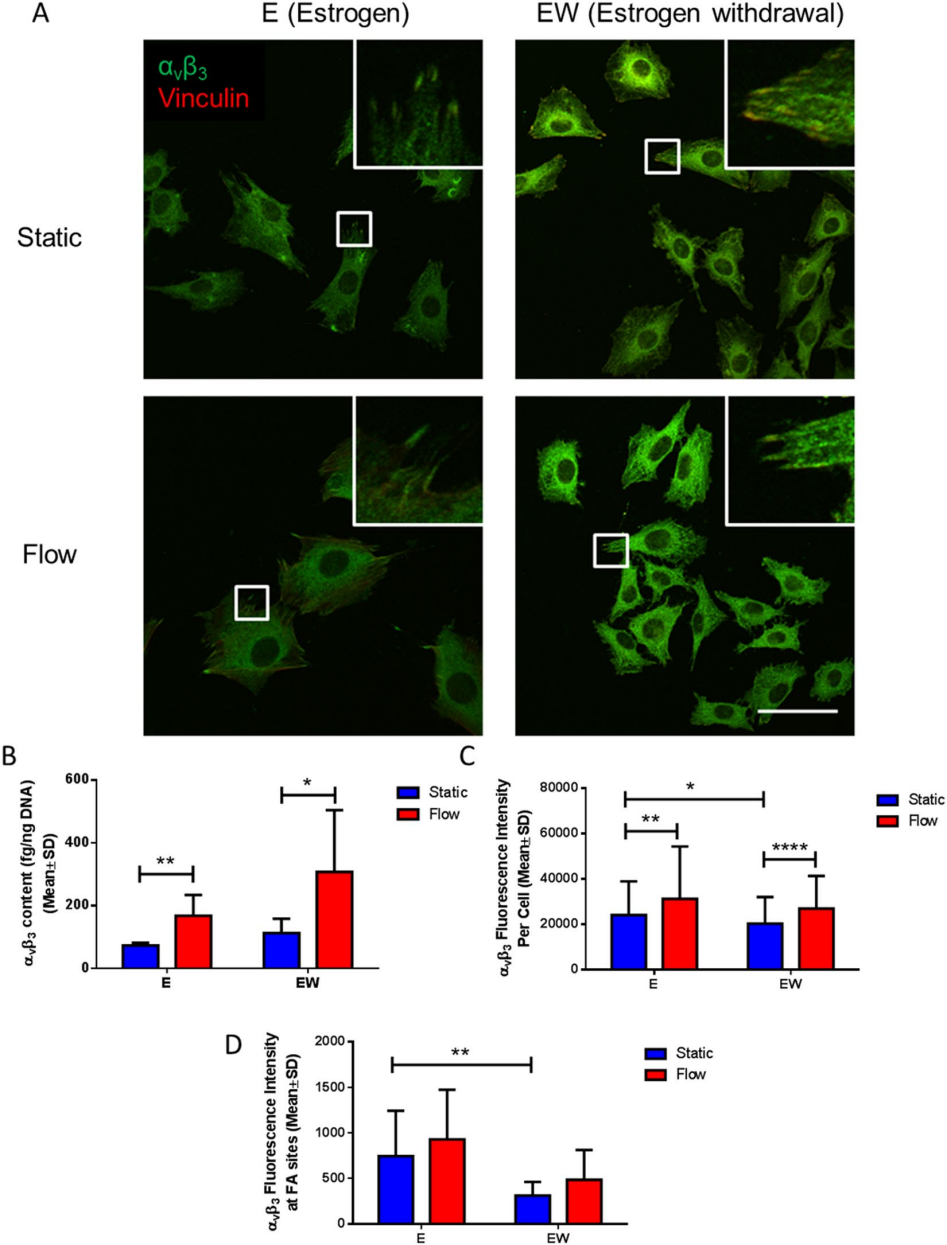
The effect of estrogen withdrawal and oscillatory fluid flow on α_v_β_3_ quantity and organisation in MLO-Y4 cells. (**A**) Immunocytochemistry images showing α_v_β_3_ and vinculin staining (N = 3, n ≥ 115 cells per group). (**B**) ELISA measurement of total α_v_β_3_ quantity normalised to DNA content (N = 6). Quantification of the images showing (**C**) α_v_β_3_ intensity per cell, and (**D**) α_v_β_3_ intensity at focal adhesion (FA) sites (Student’s t-test, *p < 0.05, **p < 0.01, ****p < 0.0001).

Given the perturbed focal adhesion assembly seen in estrogen withdrawal conditions, we next investigated the effect of estrogen withdrawal on α_v_β_3_ quantity and organisation. ELISA measurements of total α_v_β_3_ content within MLO-Y4 cells showed no statistical differences following estrogen withdrawal (p = 0.0612, N = 6) (Fig. 3B). However, analysis of immunocytochemistry imaging showed significantly lower α_v_β_3_ intensity per cell in estrogen withdrawal cells, compared to estrogen supplemented cells (p < 0.05, N = 3, n ≥ 115 cells per group) (Fig. 3C). At focal adhesion (FA) sites, this lowered α_v_β_3_ intensity in estrogen withdrawal cells was even more pronounced (p < 0.01, N = 3, n ≥ 115 cells per group) (Fig. 3D). A less ordered α_v_β_3_ organisation at FA sites was also qualitatively seen in the immunocytochemistry images for estrogen withdrawal osteocytes (Fig. 3A).

The application of oscillatory fluid flow led to higher α_v_β_3_ content in both estrogen and estrogen withdrawal groups compared to their static counterparts (p < 0.01 and p < 0.05 respectively, N = 6). This relationship was also seen in the α_v_β_3_ intensity across the whole cell area (p < 0.01 and p < 0.0001 respectively, N = 3, n ≥ 115 cells per group), but not at FA sites. Overall, these results demonstrate that estrogen withdrawal led to a lower α_v_β_3_ quantity and impaired organisation at a cell-level and, more importantly, at mechanosensitive focal adhesion sites.

### Estrogen withdrawal upregulated expression of genes associated with paracrine osteoclastogenic signalling (Rankl/Opg ratio), but perturbed the Cox-2 response to fluid shear stress

**Figure 4:**
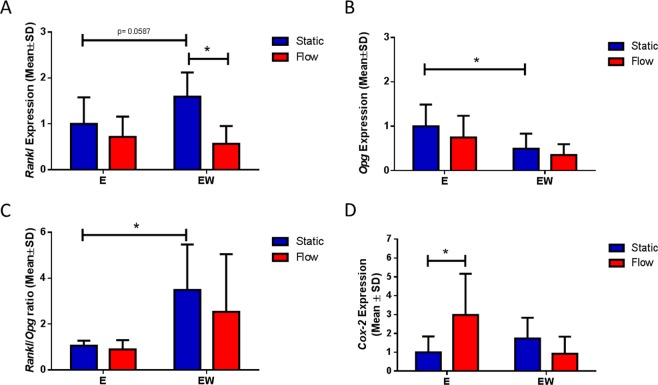
The effect of estrogen withdrawal and oscillatory fluid flow on osteoclastogenic and osteogenic signalling. RT-PCR results of (**A**) *Rankl* expression (N = 7–8), (**B**) *Opg* expression (N = 8–9), (**C**) *Rankl*/*Opg* ratio (N = 5–7), and (**D**) *Cox-2* expression (N = 7–8) (Student’s t-test, *p < 0.05).

Given the alterations in MLO-Y4 cell morphology and α_v_β_3_ organisation during estrogen withdrawal, we next investigated the effect of estrogen deficiency on genes associated with mechanotransduction (*Cox-2*) and osteoclastogenesis (*Rankl* and *Opg*). Under static culture conditions, estrogen withdrawal led to an upregulation in *Rankl* expression, in comparison to cells cultured under continuous estrogen culture, (p = 0.0587, N = 7–8), as well as a significant downregulation in *Opg* expression (p < 0.05, N = 8–9) (Fig. 4A,B). This resulted in a higher *Rankl*/*Opg* ratio in estrogen withdrawal conditions in comparison to the E group (p < 0.05, N = 5–7) (Fig. 4C).

After application of oscillatory fluid flow, Rankl expression was lower in the estrogen withdrawal group, compared to its static counterpart (p < 0.05, N = 7–8). However, there was no noticeable effect of flow on *Opg* expression, and ultimately there was no significant difference in the *Rankl*/*Opg* ratio for cells that underwent estrogen withdrawal and were mechanically stimulated (Fig. 4A–C).

For cells cultured under static conditions, estrogen withdrawal had no significant effect on *Cox-2* expression, when compared to the estrogen supplemented cells (Fig. 4D). The application of fluid shear stress led to a higher *Cox-2* response in the estrogen supplemented group, when compared to its static counterpart (p < 0.05, N = 7–8). However, this response to fluid flow was lost in estrogen withdrawal cells.

Taken together, these results demonstrate that estrogen withdrawal perturbed the normal osteogenic responses to mechanical stimulation by osteocytes, and also lead to the alteration in genes associated with paracrine osteoclastogenic signalling.

### α_v_β_3_ antagonism led to an altered cell morphology, as seen with estrogen withdrawal

Due to the fact that we showed a disorganised α_v_β_3_ organisation under estrogen withdrawal conditions, we wished to investigate whether the perturbed cell function and responses to flow following estrogen withdrawal were associated with an altered α_v_β_3_ function. Therefore, we used a small molecular inhibitor to block α_v_β_3_ function.

**Figure 5:**
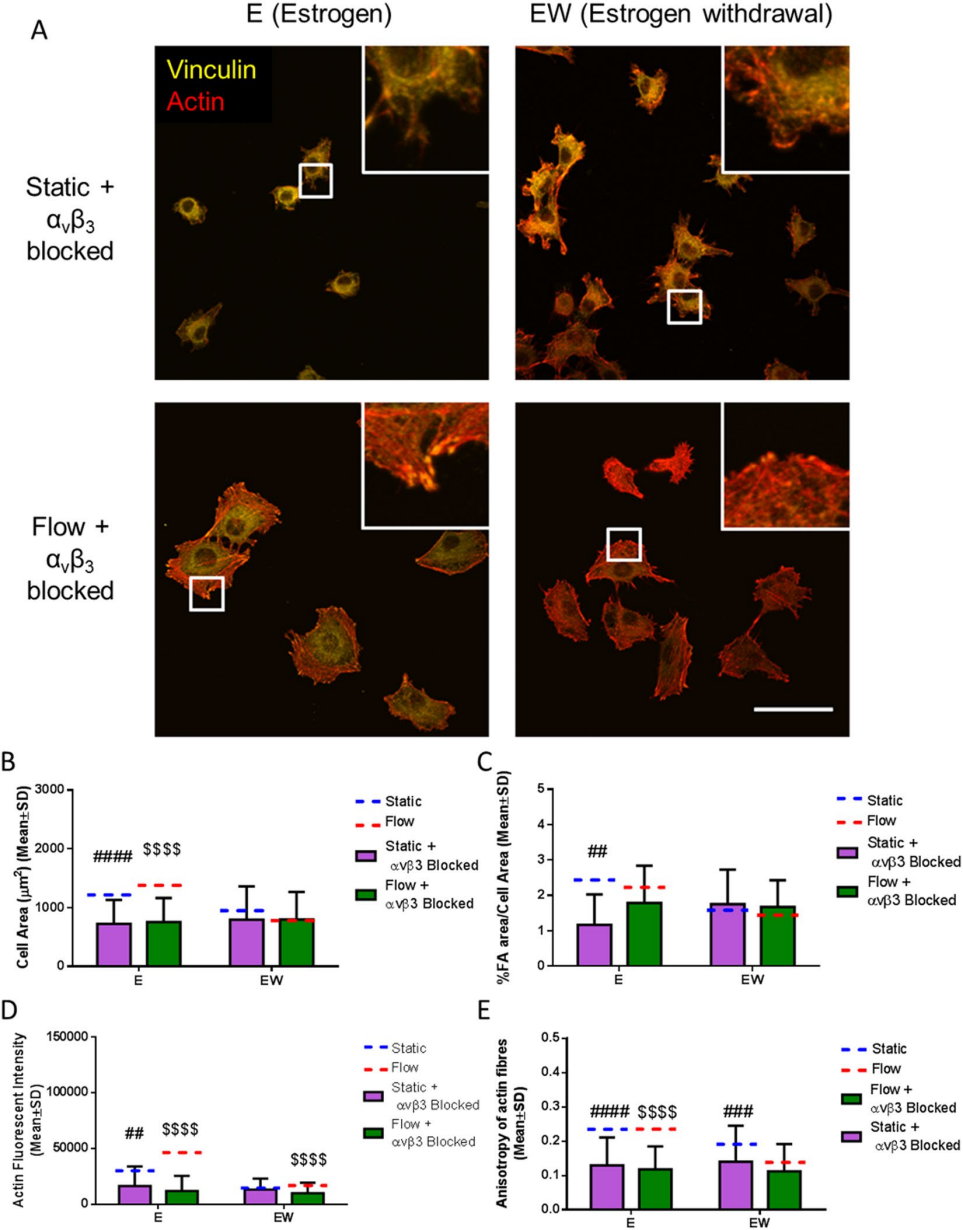
The effect of α_v_β_3_ antagonism and oscillatory fluid flow on MLO-Y4 cell morphology. (**A**) Immunocytochemistry images showing actin fibres and vinculin staining (N = 3, n ≥ 90 cells per group). Quantification of the images showing (**B**) cell area, (**C**) % focal adhesion area/cell area, (**D**) actin fluorescent intensity, and (**E**) anisotropy of actin fibres. (Student’s t-test, ^##^p < 0.01 compared to static, ^###^p < 0.001 compared to static, ^####^p < 0.0001 compared to static, ^$$$$^p < 0.0001 compared to flow).

Osteocytes that underwent α_v_β_3_ antagonism demonstrated a perturbed morphology, with a visually smaller cell area and fewer distinct focal adhesion sites visible in the estrogen group (Fig. 5A), in comparison to unblocked counterparts, see Fig. 2A. Quantification of the images confirmed a lower cell area (p < 0.0001), focal adhesion area (p < 0.01), and focal adhesion number (p < 0.05) in the estrogen cells following α_v_β_3_ antagonism (N = 3, n ≥ 90 cells per group) (Figs 5B,C and S1C). However, there was no statistical difference in cell area, focal adhesion area or number between blocked and unblocked cells in the estrogen withdrawn cells. Actin was also shown to be affected by α_v_β_3_ antagonism; whereby there was a lower actin fluorescent intensity and anisotropy in estrogen supplemented cells following α_v_β_3_ antagonism (p < 0.01, p < 0.0001, p < 0.0001 and p < 0.0001 respectively, N = 3, n ≥ 90 cells per group) (Fig. 5D,E). In estrogen withdrawal cells, α_v_β_3_ antagonism led to lower actin intensity in cells subjected to oscillatory fluid flow, and a lower actin anisotropy between the static groups (p < 0.0001 and p < 0.001 respectively, N = 3, n ≥ 90 cells per group).

A key finding was that the estrogen cells that underwent α_v_β_3_ antagonism showed a similar morphology to the estrogen withdrawal cells (unblocked). Quantitative analysis of the images confirmed no difference in cell area, focal adhesion area, or actin cytoskeleton intensity and organisation between estrogen cells following α_v_β_3_ antagonism and estrogen withdrawal cells (unblocked). These results clearly underpin the importance of α_v_β_3_ on overall cell function and implicate the dysregulation of the integrin α_v_β_3_ for the impaired cell morphology during estrogen withdrawal.

### α_v_β_3_ antagonism led to a lower α_v_β_3_ intensity per cell and at FA sites

**Figure 6:**
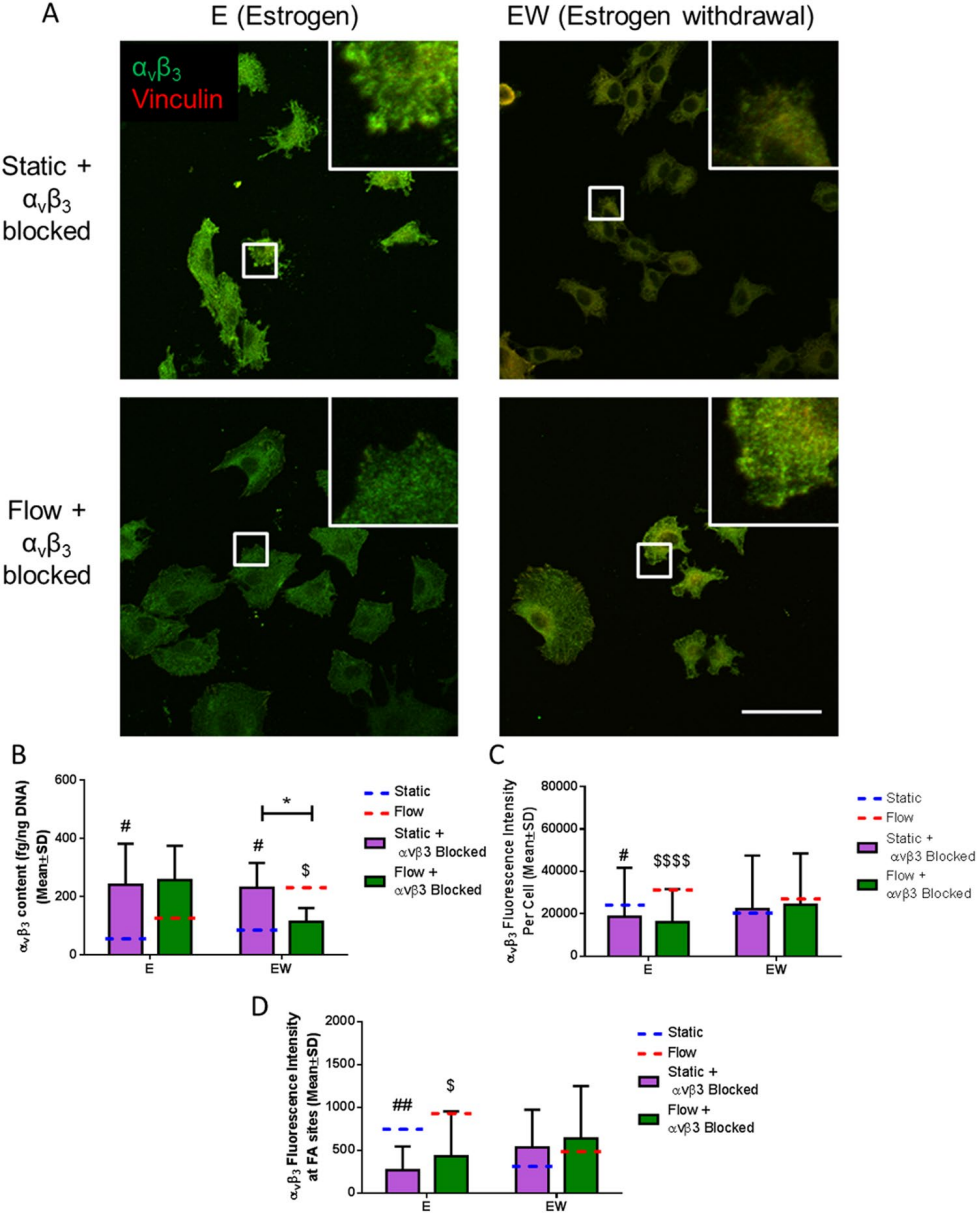
The effect of α_v_β_3_ antagonism and oscillatory fluid flow on α_v_β_3_ quantity and organisation in MLO-Y4 cells. (**A**) Immunocytochemistry images showing α_v_β_3_ and vinculin staining (N = 3, n ≥ 90 cells per group). (**B**) ELISA measurement of total α_v_β_3_ quantity normalised to DNA content (N = 6). Quantification of the images showing (**C**) α_v_β_3_ intensity per cell, and (**D**) α_v_β_3_ intensity at focal adhesion (FA) sites (Student’s t-test, *p < 0.05, ^#^p < 0.05 compared to static, ^##^p < 0.01 compared to static, ^$^p < 0.05 compared to flow, ^$$$$^p < 0.0001 compared to flow).

To confirm the efficacy of the α_v_β_3_ antagonism, we investigated the α_v_β_3_ quantity following antagonism. We investigated the intensity of α_v_β_3_ per cell and at FA sites using immunostaining. As expected, we saw a lower α_v_β_3_ intensity per cell and at FA sites in the estrogen cells in comparison to their unblocked counterparts (p < 0.05, p < 0.0001, p < 0.01, and p < 0.05 respectively, N = 3, n ≥ 90 cells per group) (Fig. 6C,D). In the estrogen withdrawal groups, we saw no change in α_v_β_3_ intensity per cell and at FA sites following antagonism, indicating that the estrogen withdrawal groups already had a lowered α_v_β_3_ quantity prior to antagonism. Further to this, there was no statistical difference in the intensity of α_v_β_3_ between α_v_β_3_ blocked estrogen cells and unblocked estrogen withdrawal cells. These results further confirm that estrogen withdrawal has a negative effect on α_v_β_3_ integrin quantity and organisation. ELISA measurements of α_v_β_3_ antagonism showed a higher α_v_β_3_ content compared to unblocked counterparts (p < 0.05, p < 0.05 and p < 0.05 respectively, N = 6) (Fig. 6B).

### α_v_β_3_ antagonism affected the Rankl/Opg response to estrogen withdrawal and resulted in a perturbed Cox-2 response to fluid flow

**Figure 7:**
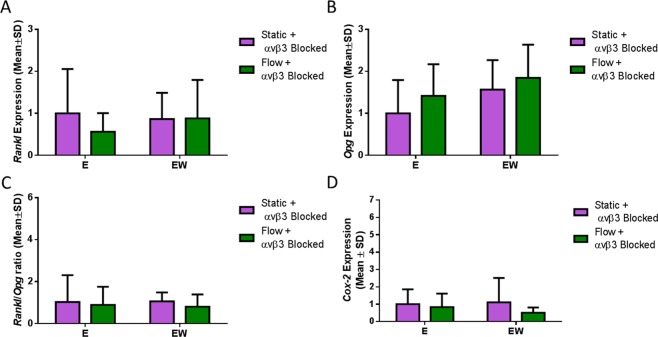
The effect of α_v_β_3_ antagonism and oscillatory fluid flow on osteoclastogenic and osteogenic signalling. RT-PCR results of (**A**) *Rankl* expression (N = 6–7), (**B**) *Opg* expression (N = 6–7), (**C**) *Rankl*/*Opg* ratio (N = 4–7) and (**D**) *Cox-2* expression (N = 6–7) (Student’s t-test, *p < 0.05).

Finally, we wished to delineate the role of the integrin α_v_β_3_ in the altered *Rankl*/*Opg* and *Cox-2* responses, seen during estrogen withdrawal. α_v_β_3_ antagonism attenuated the higher *Rankl* response and lower *Opg* response seen previously in estrogen withdrawal conditions (Fig. 7A,B) and in turn resulted in no change in *Rank*l/*Opg* ratio in estrogen withdrawal cells (Fig. 7C). This lack of *Rankl*/*Opg* response to the estrogen withdrawal conditions following antagonism may implicate the integrin α_v_β_3_ as an important transducer of estrogen-related signalling. Moreover, following α_v_β_3_ antagonism, the *Cox-2* response to flow was absent in all groups (Fig. 7D). Taken together, these results show the importance of changes in the localisation of the integrin α_v_β_3_ in the estrogen deficient conditions of post-menopausal osteoporosis.

## Discussion

**Figure 8:**
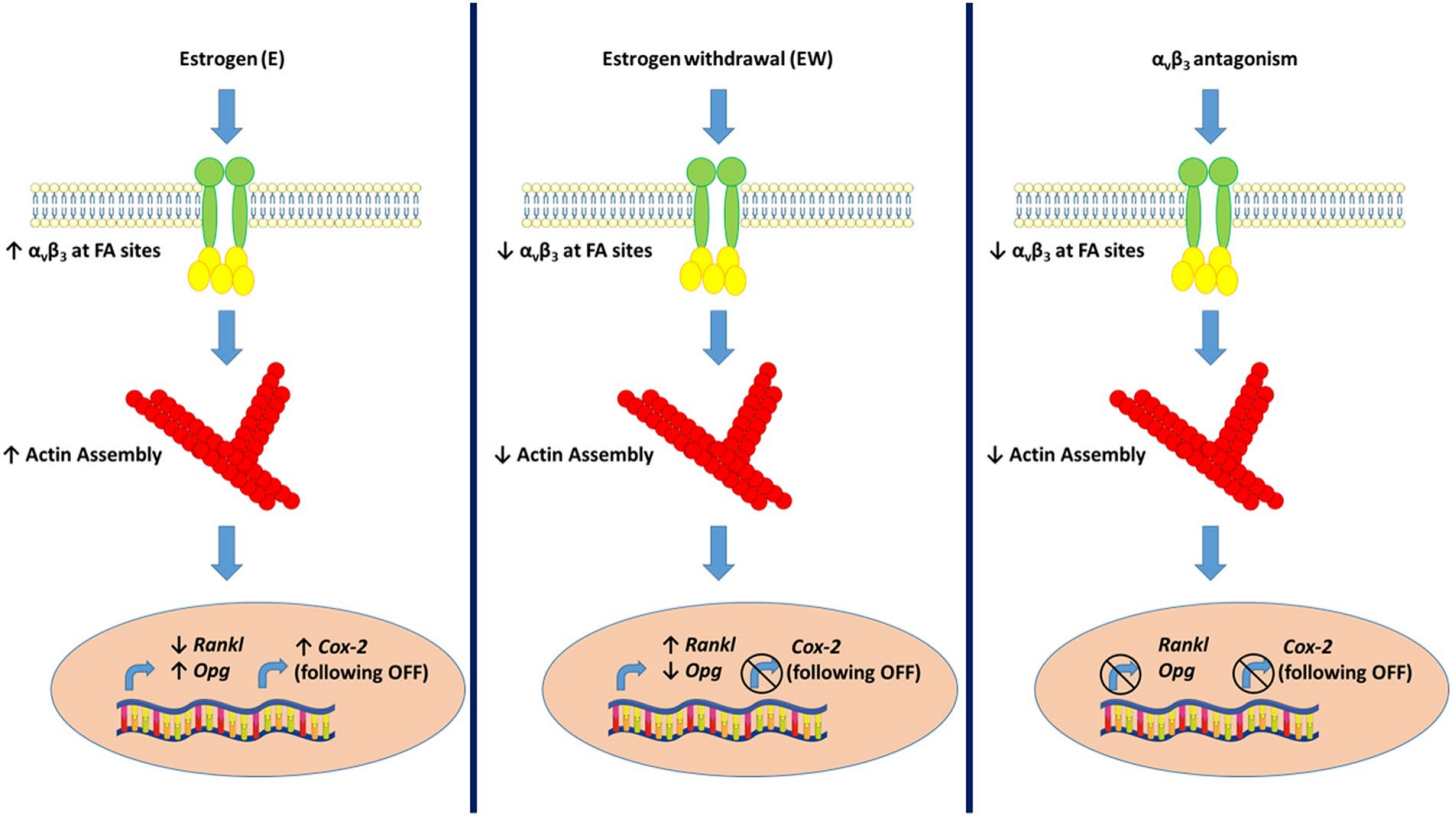
A schematic depicting the effect of estrogen treatment (E), estrogen withdrawal (EW), and α_v_β_3_ antagonism.

Given the importance of the integrin α_v_β_3_ in osteocyte mechanotransduction, we sought to investigate the effect of estrogen withdrawal on the integrin α_v_β_3_ and α_v_β_3_-mediated signalling. Here, we report for the first time that, under static conditions, cell area, focal adhesion area, and the distribution of α_v_β_3_ at mechanosensitive focal adhesion sites were all negatively affected by estrogen withdrawal conditions. Moreover, downstream mechanotransduction signalling associated with the integrin α_v_β_3_ (*Cox-2* gene expression) was negatively affected following estrogen withdrawal. Osteocytes, in static conditions, that underwent estrogen withdrawal also increased expression of the *Rankl* gene and downregulated *Opg* expression, which are known to regulate osteoclastogenesis in a paracrine fashion. To investigate whether the integrin α_v_β_3_ was responsible for the changes seen in estrogen withdrawal conditions, we blocked the integrin α_v_β_3_ with an antagonist. Most interestingly, we report similar changes in cell morphology and downstream signalling in the absence of functional α_v_β_3_ mechanoreceptors, as we had observed for cells that underwent estrogen withdrawal. Therefore, our results implicate a link between the estrogen withdrawal conditions of post-menopausal osteoporosis and altered α_v_β_3_ functionality (Fig. 8).

Previous studies have clearly demonstrated defective osteocyte mechanotransduction during estrogen withdrawal *in vitro*^39,42^. *In vivo*, the number of β_3_-containing bone cells in cortical bone was lower compared to controls in an estrogen deficient rat model of post-menopausal osteoporosis^42^. Here, we demonstrate an altered osteocyte morphology, specifically by means of a lower cell area and altered actin cytoskeleton, following estrogen withdrawal, which is in keeping with previous research^39^. We report for the first time impaired focal adhesion assembly in cells that are cultured under estrogen withdrawal conditions, along with a lower α_v_β_3_ intensity at these compromised focal adhesion sites. Disperse cytosolic staining of vinculin was clearly visible in estrogen withdrawal cells. However, only distinct focal adhesion sites were quantified. The α_v_β_3_ staining in our estrogen treated cells showed similarities to studies of MLO-Y4 cells in culture media (without estrogen supplementation)^15,45^, but the spatial relationship of α_v_β_3_ to other FA proteins, such as vinculin, has not been previously investigated. Recently, β_3_ integrins were found along mouse osteocyte cells processes *in vivo* within specialised structures containing pannexin1, P2X7R, and CaV3.2-1. However, in that study the larger vinculin containing-focal adhesion complexes were found around the cell body and not the tightly packed cell processes, due to space constraints^22^. In our *in vitro* study, cells do not have space constraints, and we show that vinculin co-localises with α_v_β_3_ integrin. While it is known that the β_3_ integrin binding sites along osteocyte cell processes are key sites in osteocyte mechanotransduction *in vivo*, particularly due to strain amplification, computational modelling has shown that osteocytes subjected to fluid shear stress *in vitro*, as seen in in this study, do transduce this mechanical stimulation through integrin binding sites found along the cell processes and cell body^46^. As such, we have measured focal adhesion sites found along the cell processes and cell body in our study.

Together our findings of an increase in *Rankl*/*Opg* ratio, under static conditions, and abrogation of the *Cox-2* response to oscillatory fluid flow demonstrate an altered response to mechanical stimulation in estrogen withdrawal conditions. RANKL is a cytokine produced by osteocytes, which binds to RANK receptors on osteoclast progenitors and regulates osteoclast differentiation and activity, whereas OPG is also produced by osteocytes but acts a decoy receptor for RANKL^47–51^. It has been established that the application of oscillatory fluid shear stress to MLO-Y4 cells results in a decrease in the *Rankl*/*Opg* ratio^16^, which indicates that mechanically stimulated osteocytes reduce paracrine signalling of osteoclastogenesis. We showed that estrogen withdrawal led to an increased *Rankl*/*Opg* ratio. Estrogen withdrawal has been shown to induce osteocyte apoptosis^41,52^, which, in turn, leads to changes in RANKL expression^53^. Interestingly, it has previously been reported that in osteoblasts, estrogen treatment led to higher *Opg* expression in a dose-dependent manner, compared to untreated control cells^31^. COX-2 is an enzyme that produces prostaglandins to promote inflammation, and expression of the *Cox-2* is upregulated in osteocytes following oscillatory fluid flow^2,16^. Given the importance of *Cox-2* for normal fracture repair^54^ and an increased *Rankl*/*Opg* ratio for inducing osteoclast activity^7,9,26^, we propose that under estrogen withdrawal, osteocyte signalling would contribute to the increased bone resorption seen in post-menopausal osteoporosis^26,55^.

The importance of integrin α_v_β_3_ in osteocyte mechanotransduction has been recently demonstrated^15,17^, and, in keeping with a previous study^15^, our results show that α_v_β_3_ antagonism resulted in a smaller cell area. Interestingly, α_v_-integrin antagonism in melanoma cells was shown to lead to rearrangement and disassembly of focal adhesion proteins^56^ and we propose that integrin antagonism may have a similar effect on focal adhesion in osteocytes. It is interesting to note that while focal adhesion area was lower following α_v_β_3_ antagonism, some distinct focal adhesion sites were present. If we assume that the antagonist blocked all α_v_β_3_-containing focal adhesion sites, it is likely that these remaining focal adhesion sites contained other integrin subunits, such as β_1_ integrins^4^. α_v_β_3_ antagonism resulted in a lower α_v_β_3_ fluorescent intensity. Moreover, α_v_β_3_ antagonism resulted in an attenuated *Rankl* and *Opg* response following estrogen withdrawal. As the integrin α_v_β_3_ was shown to mediate OPG protein production in a human endothelial-breast cancer cell co-culture^57^, we propose that the integrin α_v_β_3_ plays a similar role in regulating *Rankl* and *Opg* signalling in osteocytes. Previous research studying the role of α_v_β_3_ in *Rankl* and *Opg* expression, saw no significant changes following fluid flow, and therefore it was unclear what role the integrin α_v_β_3_ played in the *Rankl*/*Opg* response to flow in this instance^15^. Following α_v_β_3_ antagonism, we saw a diminished *Cox-2* response to oscillatory fluid flow. Abrogation of this *Cox-2* response to flow following α_v_β_3_ antagonism has been demonstrated previously for MLO-Y4 cells cultured under control conditions (no added estrogen)^15^. Other integrin subunits, notably β_1_ integrins, play an important role in osteocyte mechanotransduction and have been shown to be necessary for normal *Cox-2* response to flow^16,58^. While the role of β_1_ integrins was not investigated in this study, it is interesting to note that β_1_ integrins were not fully able to recover *Cox-2* responses to flow following α_v_β_3_ antagonism. This may indicate that β_1_ and β_3_ integrins are closely linked in function *in vitro*. By showing an altered focal adhesion assembly, α_v_β_3_ localisation at FA sites, and abrogated *Cox-2* response to flow following estrogen withdrawal or α_v_β_3_ antagonism, our study is the first to demonstrate a link between altered osteocyte mechanobiology during estrogen withdrawal and altered α_v_β_3_ functionality.

Following mechanical stimulation of osteocytes, α_v_β_3_ has been shown to activate MAP kinase pathways leading to an upregulation in c-fos, IGF-1, and *Cox-2*^45^. It has been proposed that upregulation of c-fos is associated with the Ca^2+^ influx pathway^45^, which has separately been shown to be mediated by α_v_β_3_^17^. In osteoblasts, the α_v_β_3_ pathway has been studied more extensively, with the application of fluid flow leading to a synergistic activation of FAK and shc, resulting in the activation of PI3-K and Akt/mTOR/p70S6K pathway, and ultimately increases in *Cox-2* expression^14^. In osteocytes, FAK, PI3-K, and Akt have been shown to be activated in response to fluid shear stress, but it is unknown whether this is mediated by α_v_β_3_^59^. Estrogen treatment has been shown to induce FAK phosphorylation, and activation of PI3-K in breast cancer cells^60^ and in HUVECs^61^. The breast cancer cells also activated Akt and formed focal adhesion sites in response to estrogen treatment^60^. Thus we propose that *Cox-2* expression by osteocytes following oscillatory fluid flow may arise because α_v_β_3_ integrins activate FAK, PI3-K and Akt pathways. Moreover, due to the role of estrogen in regulating FAK and PI3-K pathways in other cell types, we propose that estrogen withdrawal may result in a defect in this proposed α_v_β_3_ pathway and could account for the abrogated *Cox-2* response to fluid flow.

There are a number of limitations that must be considered. Firstly, this study involved the use of MLO-Y4 osteocyte-like cells, which were cultured in a monolayer within a parallel plate flow bioreactor. While this cannot fully mimic the mechanical stimulation of osteocytes within the lacunar-canalicular system *in vivo*, both the MLO-Y4 cells and the bioreactor have been used a model extensively *in vitro* to understand the mechanobiology of osteocytes during health and disease^15,16,39,62–64^. Secondly, our regime involved immediate withdrawal of estrogen, but the exact timeline of changes in serum estradiol levels during the menopause *in vivo* is unknown. In humans, serum estradiol levels deplete over a four year period^65^, whereas in mice, serum estradiol levels were shown to be lower than normal controls one week after ovariectomy^66^. Thirdly, it was surprising that ELISA measurements of α_v_β_3_ antagonism showed a higher α_v_β_3_ content compared to unblocked counterparts. We believe that these higher readings may be due to the fact that the α_v_β_3_ antagonist was detected by the ELISA along with MLO-Y4 α_v_β_3_ integrins. However, it should be noted that the immunocytochemistry staining confirmed the effectiveness of the α_v_β_3_ antagonist.

Our results show that the mechanosensor, integrin α_v_β_3_, and downstream gene expression is negatively affected by estrogen withdrawal and therefore highlights the importance of integrin α_v_β_3_ in post-menopausal osteoporosis. As β_3_ integrin expression is affected by estrogen deficiency in OVX animals, this study implicates estrogen withdrawal as the mechanism responsible for altered α_v_β_3_ expression and resultant downstream signalling in osteocytes during post-menopausal osteoporosis. Further study to determine a means of protecting the normal expression and signalling of these integrins may offer a potential novel therapy for post-menopausal osteoporosis. Given the altered morphology and gene expression in both estrogen withdrawal conditions and following α_v_β_3_ antagonism, it is clear that the integrin α_v_β_3_ is affected by estrogen withdrawal and this in turn affects α_v_β_3_-mediated signalling. However, it is unlikely that the integrin α_v_β_3_ is the only mechanosensor affected by estrogen withdrawal. Many other mechanosensors including β_1_ integrins^16^, the primary cilium^2^, and stretch activated ion channel, TRPV4^3^, have all been shown to be important in osteocyte mechanotransduction. To date, the effect of estrogen withdrawal on the ability of these other mechanosensors to transduce mechanical stimulation has not been reported. Given the multitudinous effects of estrogen on cell function, studying the effects of estrogen withdrawal on these other osteocyte mechanosensors may provide an exciting avenue of future research.

## Conclusions

Our results show that osteoporotic conditions induced by estrogen withdrawal negatively affected mechanosensor function, specifically through alterations in α_v_β_3_ quantity at focal adhesion sites, and that also resulted in impaired mechanobiological responses to flow (*Cox-2* and *Rankl*/*Opg* gene expression). These altered responses to flow were also seen following α_v_β_3_ antagonism. We propose that due to the estrogen deficient conditions of post-menopausal osteoporosis, osteocytes undergo altered α_v_β_3_ organisation and resultant changes in downstream signalling, particularly those that govern osteoclastogenesis. This research thus uncovers a previously unknown element of the bone loss cascade, whereby estrogen deficiency alters the mechanosensory ability of osteocytes and they in turn contribute to paracrine signalling to govern osteoclast resorption.

## Materials and Methods

### Cell Culture, and Estrogen Treatment Regimes

MLO-Y4 mouse osteocyte-like cells were cultured on type I collagen (0.15 mg/ml in 0.02 M acetic acid and phosphate buffered saline (PBS)) coated T-75 flasks in α-minimum essential media (α-MEM) supplemented with 5% fetal calf serum (FCS), 5% fetal bovine serum (FBS), 2 mM L-glutamine, 100 U/mL penicillin, and 100 µg/mL streptomycin at 37 °C in a humidified environment at 5% CO_2_. The effect of estrogen treatment and estrogen withdrawal on MLO-Y4 cells was studied using the following groups: (1) standard culture media with no added 17β-estradiol (Ctrl), (2) continuous treatment with 10 nM 17 β-estradiol for 5 days (E), and (3) pre-treatment with 10 nM 17β-estradiol for 3 days and withdrawal for 2 further days (EW), following previous approaches developed in our laboratory^39^. On day 3 (two days prior to the induction of oscillatory fluid flow/static conditions), cells were seeded onto collagen coated glass slides, at a density of 200,000 cells/slide, and cultured for two days in accordance with their treatment groups.

### Integrin α_v_β_3_ Antagonism

The integrin α_v_β_3_ was blocked using a small molecule inhibitor for α_v_β_3_ integrins, IntegriSense 750 (PerkinElmer)^15,17^. After removal of culture medium, 1 mL of media containing 0.5 µM IntegriSense 750 was added to each slide for 30 min prior to flow/static conditions. Following this, slides were washed twice with PBS.

### Oscillatory Fluid Flow

Laminar oscillatory fluid flow was applied to a cell monolayer using a parallel plate bioreactor system, which consisted of a syringe pump (NE-1600, New Era Pump Systems, Farmingdale, NY), polycarbonate plate chambers, media reservoirs connected by gas-permeable and platinum-cured silicone tubing (Cole-Parmer, Vernon Hills, IL)^15,39^. The fluid flow regime subjected the cell monolayer to a shear stress of 1 Pa at 0.5 Hz for 1 h, which is within the range of shear stresses experiences by osteocytes *in vivo*^25,67–70^. For comparison, static counterparts in each treatment group were not subjected to laminar oscillatory fluid flow conditions for the same time-period.

### DNA Content

DNA content was measured using a Hoechst assay. Briefly, 1 mL of sterile deionised (DI) water was carefully placed on each slide and the cells were lysed via the freeze-thaw lysis method in a −80 °C freezer. DNA samples were added to wells with the working dye solution consisting of Hoechst dye: 1X Hoechst Buffer (1:1,000) (Sigma Aldrich). The fluorescence intensity was read using a spectrophotometer for an excitation of 360 nm and at an emission of 460 nm. Calf thymus DNA (Sigma Aldrich) was used as a standard.

### Integrin α_v_β_3_ Quantification

α_v_β_3_ integrin concentration was quantified by means of an α_v_β_3_ ELISA (Emelca Bioscience) as per the supplier’s protocol. Briefly, cells were lysed used the freeze-thaw lysis method, as above. The sample lysate was placed in the ELISA wells and an antibody against α_v_β_3_ was added to each well. Biotin labelled antibodies and horseradish peroxidase were added to further label the samples for α_v_β_3_ integrins. The well plate was incubated for 37 °C and washed with a buffer between each of these steps, according to the supplier’s protocol. Reagents to aid in colour visualisation were added and the plate absorbance was read at 450 nm using a spectrophotometer (Synergy HT, BioTek). α_v_β_3_ integrin concentration readings were normalised to DNA content.

### Immunofluorescence

Immunofluorescence was used to study and quantify the spatial distribution of α_v_β_3_ integrins in cells from each treatment group cultured statically or following application of flow. Briefly, the cells were washed in PBS and fixed in 3.8% formaldehyde solution, and then permeabilised in 0.1% Triton-X. The cells were incubated in 10% BSA to prevent non-specific binding occurring during the staining process. Integrin α_v_β_3_ staining was performed using an anti-α_v_β_3_ antibody directly conjugated to Alexa Fluor® 488 (1:100) (Santa Cruz). Vinculin staining was performed using a mouse anti-vinculin primary antibody (1:800) (Sigma Aldrich) and goat anti-mouse Alexa Fluor® 647 secondary antibody (1:200) (Abcam) to label focal adhesion and facilitate the investigation of α_v_β_3_ integrin co-localisation (See Supplementary Table 1 for further information on antibodies used). The cells were also stained with TRITC (Tetramethyl Rhodamine Iso-Thiocyanate) Phalloidin and DAPI (4′,6-diamidino-2-phenylindole) to facilitate imaging of the actin cytoskeleton and nucleus respectively. Z-stack imaging was done using a Fluoview FV100 confocal laser scanning microscope system (Olympus) at a magnification of 60x (oil immersion) with a step size of 0.5 µm.

All image analysis was completed using ImageJ software^71^. The z-stacks of the images taken were combined as maximum intensity projections and these combined images were used for all image analysis. Cell area and overall actin fluorescence intensity were measured using the actin stained images. Cell area was measured by thresholding the images to remove background fluorescence and then using the “wand tool” to select the region of interest around each cell. Where two cells were touching, the region of interest was drawn manually with the “freehand tool”. Anisotropy of the actin fibrils was determined using a ImageJ plugin, known as FibrilTool^72^. The vinculin stained images were used to identify distinct focal adhesion sites across the entire cell. Identification of distinct focal adhesion sites was enabled by means of a previously published semi-automatic protocol^73^. Focal adhesion area was normalised to cell area. The focal adhesion ROIs thresholded in this process were then used to determine the intensity of the α_v_β_3_ staining at focal adhesion sites. All fluorescent intensities were measured using the integrated density of each cell/ROI with their corresponding background integrated density subtracted.

### Real Time PCR

Relative gene expression was studied by quantitative Real Time Polymerase Chain Reaction (qRT-PCR). The genes of interest included *Cox-2*, *Rankl*, and *Opg*, with *Rpl13A* used as a reference gene (Supplementary Table 2). RNA was isolated using Qiagen RNeasy kits as per manufacturer’s instructions. RNA purity and yield were assessed using a spectrophotometer (DS-11 FX, DeNovix), with 260/280 ratios of >1.9 for all samples. 250–500 ng of RNA was then transcribed into cDNA using Qiagen Quantinova reverse transcription kits and thermal cycler (5PRIMEG/O2, Prime). qRT-PCR was carried out with a Qiagen Quantinova SYBR Green PCR kit and a StepOne Plus PCR machine (Applied Biosciences). Analysis of the results was done using the Pfaffl method^74^.

### Statistical analysis

Data is presented as mean ± standard deviation. Statistical significance was determined by means of unpaired two-tailed Student’s t-tests. All statistical analyses were performed using GraphPad Prism version 6 (Windows, GraphPad Software, La Jolla California USA, www.graphpad.com) and p-value of 0.05.

## Supplementary information

Supplementary Information

## Data Availability

The datasets generated during and/or analysed during the current study are available from the corresponding author on reasonable request.
