# Supplementary material for: Estrogen deficiency impairs integrin αvβ3-mediated mechanosensation by osteocytes and alters osteoclastogenic paracrine signalling
Source: Sci Rep. 2019 Mar 15;9:4654. doi: 10.1038/s41598-019-41095-3 (PMC6420496; doi:10.1038/s41598-019-41095-3)
Supplement: Supplementary file 1 — Supplementary Information [file 41598_2019_41095_MOESM1_ESM.pdf]

Estrogen deficiency impairs integrin $\alpha_v\beta_3$-mediated mechanosensation by osteocytes and alters osteoclastogenic paracrine signalling

Ivor Geoghegan[1,2], David A. Hoey[2,3,4,5], *Laoise M. McNamara[1,2]

[1]Mechanobiology and Medical Device Research Group (MMDRG), Biomedical Engineering, National University of Ireland, Galway, Ireland

[2]Centre for Research in Medical Devices (CÚRAM), National University of Ireland, Galway, Ireland

[3]Trinity Centre for Bioengineering, Trinity Biomedical Sciences Institute, Trinity College Dublin, Ireland

[4]Dept. of Mechanical and Manufacturing Engineering, School of Engineering, Trinity College Dublin, Ireland

[5]Advanced Materials and Bioengineering Research Centre, Trinity College Dublin & RCSI, Dublin 2, Ireland

*Address for correspondence:

Laoise M. McNamara, Ph.D.

Department of Mechanical and Biomedical Engineering

National University of Ireland Galway

Galway,

Ireland

Phone: (353) 91-492251

Fax: (353) 91-563991

Email: Laoise.McNamara@nuigalway.ie

Supplementary Results

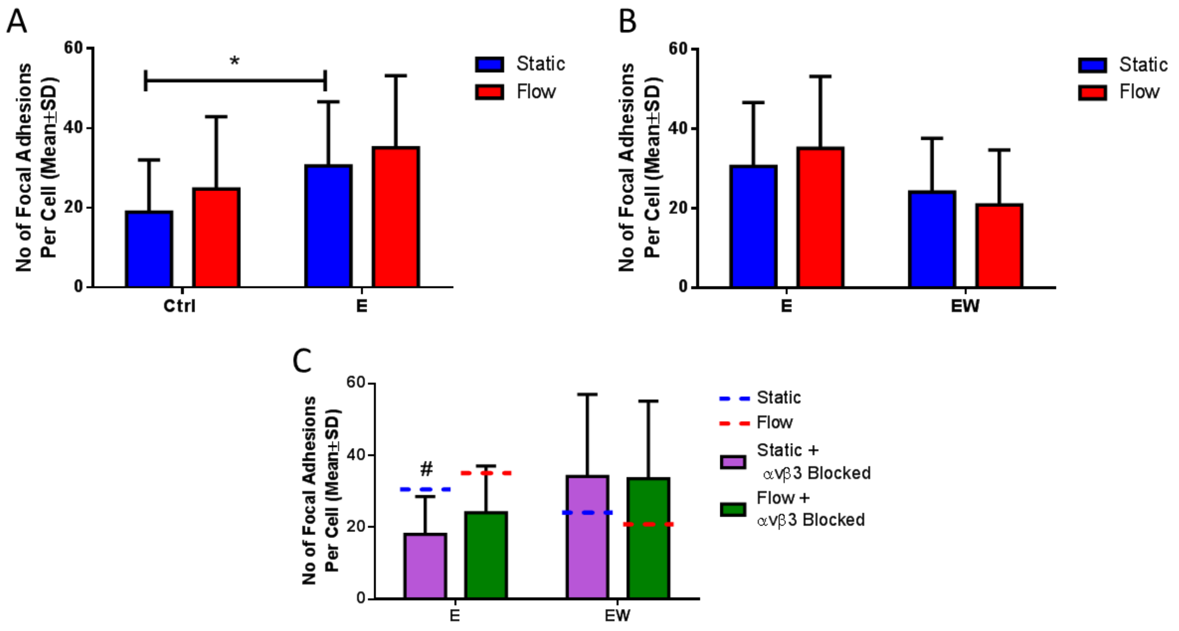

Supplementary Figure 1: Number of focal adhesions per MLO-Y4 cell (N=3, n≥90 cells per group). Quantification of the vinculin stained images showing the effect of (A) estrogen treatment, (B) estrogen withdrawal, and (C) $\alpha_v\beta_3$ antagonism on the number of focal adhesions per cell following static or fluid flow conditions (Student's t-test, *$p<0.05$, #$p<0.05$ compared to static)

Supplementary Methods

Supplementary Table 1: List of antibodies used

Antibody	Supplier	Reference	Dilution
Integrin $\alpha_v\beta_3$	Santa Cruz	sc-7312 AF488	1:100
Vinculin	Sigma Aldrich	V9131	1:800
goat anti-mouse Alexa Fluor® 647 secondary antibody	Abcam	ab150115	1:200

Supplementary Table 2: Primers used for qRT-PCR analysis

Gene	Sequence	Tm used (°C)	Primer Concentration	Amplicon size
Opg F	GCCACGCAAAAGTGTGGAAT	57.5	700 nM	123 bp
Opg R	TTTGGTCCCAGGCAAACTGT			
Rankl F	CCCATCGGGTTCCCATAAAG	58	700 nM	140 bp
Rankl R	AGCAAATGTTGGCGTACAGG			
Cox-2 F	CTGGAACTGGAACATGGACTCACTCAGTTT	62	700 nM	109 bp
Cox-2 R	AGGCCTTTGCCACTGCTTGTA			
Rpl13a F	TACCAGAAAGTTTGCTTACCTGGG	57.3	700 nM	151 bp
Rpl13a R	TGCCTGTTTCCGTAACCTCAAG			
